# Congenital Diaphragmatic Hernia and Joint Laxity: A Putative Link with Heritable Connective Tissue Disorders

**DOI:** 10.3390/genes16091066

**Published:** 2025-09-10

**Authors:** Alessandra Di Pede, Monia Magliozzi, Laura Valfré, Maria Lisa Dentici, Flaminia Pugnaloni, Viola Alesi, Andrea Conforti, Irma Capolupo, Annabella Braguglia, Andrea Dotta, Pietro Bagolan, Antonio Novelli, Maria Cristina Digilio

**Affiliations:** 1 Medical and Neonatal Sub-Intensive Unit, Bambino Gesù Children’s Hospital IRCCS, 00165 Rome, Italy; alessandra.dipede@opbg.net (A.D.P.); annabella.braguglia@opbg.net (A.B.); 2 Laboratory of Medical Genetics, Translational Cytogenomics Research Unit, Bambino Gesù Children’s Hospital IRCCS, 00165 Rome, Italy; monia.magliozzi@opbg.net (M.M.); marialisa.dentici@opbg.net (M.L.D.); viola.alesi@opbg.net (V.A.); antonio.novelli@opbg.net (A.N.); 3 Neonatal and Pediatric Surgery Unit, Bambino Gesù Children’s Hospital IRCCS, 00165 Rome, Italy; laura.valfre@opbg.net (L.V.); andrea.conforti@opbg.net (A.C.); pietro.bagolan@opbg.net (P.B.); 4 Neonatal Intensive Care Unit, Bambino Gesù Children’s Hospital IRCCS, 00165 Rome, Italy; irma.capolupo@opbg.net (I.C.); andrea.dotta@opbg.net (A.D.); 5 Medical Genetics Unit, Translational Pediatrics and Clinical Genetics Research Area, Bambino Gesù Children’s Hospital IRCCS, 00165 Rome, Italy; mcristina.digilio@opbg.net

**Keywords:** congenital diaphragmatic hernia, joint laxity, connective tissues disorders, Marfan syndrome

## Abstract

**Background/Objectives:** The etiology of congenital diaphragmatic hernia (CDH) remains unknown in over 50% of cases, although multiple heterogeneous causative defects have been identified. Emerging evidence suggests that specific genes and molecular pathways involved in connective tissue biology may contribute to CDH development. Associations between CDH and connective tissue disorders have been reported, including cases in Marfan syndrome and a prevalence of CDH in 34% of patients with arterial tortuosity syndrome. Noticing joint laxity in several CDH patients, we aimed to investigate the presence of genetic variants linked to connective tissue disorders in this subgroup, focusing on patients enrolled in the follow-up program at Bambino Gesù Children’s Hospital. **Methods**: We selected patients diagnosed with CDH who also exhibited joint laxity based on a positive Beighton scale. These individuals underwent molecular analysis targeting genes known to be associated with heritable connective tissue disorders. **Results:** Genetic testing revealed variants in several genes across our patient series. These included mutations in *FBN1*, *FBN2*, *ZNF469*, *VEGFA*, *NOTCH1*, *ELN*, *MCTP2*, and *SMAD6*. In some cases, the variants were inherited paternally, while others appeared de novo. Most of these variants were classified as of unknown significance according to ACMG guidelines. **Conclusions:** (1) Several “variants of unknown significance” in different genes causative for connective tissue disorders have been detected in half of the present series of patients with CDH and joint laxity; (2) although the majority of the variants are classified accordingly to the ACMG as “variants of unknown significance”, a role of predisposition or susceptibility to CDH cannot be excluded; (3) a precise clinical evaluation for features of connective disorders should be recommended in the diagnostic workflow of patients with CDH.

## 1. Introduction

Congenital Diaphragmatic Hernia (CDH) is a life-threatening congenital abnormality affecting diaphragm and lung development with a variable defect size in the diaphragm, often associated with pulmonary hypoplasia, postnatal pulmonary hypertension, and cardiac disfunction [1]. CDH is an emergency requiring surgery after birth. Diaphragmatic eventration or significant thinning of the diaphragm is considered to be a related birth defect that may share a similar etiology and that may occur during the development of the human diaphragm between the 4th and 12th week of gestation. There are several different types of diaphragmatic hernia: the commonest defect is located in the posterolateral diaphragm (Bochdalek hernia), while hernias involving the anterior portion of the diaphragm (Morgagni hernia) are less frequent [2].

The incidence of CDH is 1.7–5.7 per 10,000 live-born infants [3]. Despite advances in neonatal care, the overall mortality rate for CDH remains high. Prenatal diagnosis is possible by an ultrasound examination performed during the second trimester in most affected infants. CDH may occur as an isolated defect (simplex CDH) in 40–50% of patients or can be associated with extra-diaphragmatic malformations including cerebral (5–75% patients), cardiovascular (4–63% patients), genitourinary (5–27% patients), and gastrointestinal (1–20% patients) anomalies [4,5].

The mortality rate of isolated CDH is 10–20% lower than in syndromic CDH patients [6].

The genetic etiology of CDH is highly heterogeneous, including chromosomal anomalies, copy number variants (CNVs), and single gene defects [6,7,8,9,10,11,12,13,14]. Evidences are arising on the connection between certain genes and pathways that play a role in the development of CDH. Different single genes have been implicated in the etiology of CDH and they have been identified by characterization of mutant mouse models and through the analysis of recurrent chromosomal anomalies in CDH patients. Different single genes have been described in the etiology of CDH, including transcription factor *GATA 4* [12].

In regard to heritable connective disorders, several single reports of Marfan syndrome (MFS) with CDH have been described in the literature [10,11], and CDH is reported in 34% of the patients with arterial tortuosity syndrome [15] and in several reports of LTBP4-cutis laxa [16]. Nevertheless, CDH is not yet listed among clinical criteria in the Ghent nosology of MFS [17,18]. Additionally, diagnosis of CDH has been recently reported in patients with biallelic *TGFBR1*-related Loeys Dietz syndrome [19] and in other rare genes connected to heritable connective tissue disorders with marfanoid habitus [20] or Ehlers-Danlos phenotype [21].

Following the observation of joint laxity in several patients, we set up a study to perform molecular analysis for genes involved in the etiology of heritable connective tissue disorders in patients with CDH associated with positive Beighton scale [22] referred to Bambino Gesu’ Children Hospital follow-up program.

## 2. Materials and Methods

### 2.1. Patients and Clinical Methods

We included in this study 124 pediatric patients with CDH surgically corrected included in the follow-up program of our hospital evaluated from January 2016 to December 2023. All our CDH patients, after hospital discharge, undergo a scheduled clinical multidisciplinary evaluation at 6 and 12 months of age, followed by a yearly follow up at the Rare Disease Department since January 2014. This program includes pediatric, psychological, and physiotherapic evaluation, 2-dimensional color-Doppler echocardiography, renal ultrasound examination, and ophthalmological and audiometric examination. Additionally, a clinical genetic evaluation by expert dysmorphologists in all CDH patients is planned. Beighton score was assessed [22], considering the 0–3 score as negative and 4–9 as positive for joint laxity [23]. Details about CDH side and defect size, patch repair, and PH and PDA presence were recorded from clinical and surgical records.

### 2.2. Genetic Analysis

Genetic testing was performed at the Genetic Laboratories of the Bambino Gesù Chilren’s Hospital, IRCCS. Standard chromosomal analysis and comparative genomic Hybridization (CGH) array were performed in all patients after gaining informed consent for genetic analysis. Patients with Beighton score 4–9 underwent NGS panel testing for heritable connective tissue disorders, including fibrillinopathies, collegenopathies, and aortopathies.

Genomic DNA from peripheral blood was extracted by platform QIAsymphony AS (Qiagen, Hilden, Germany) according to standard manufacturer protocol. Chromosomal Microarray Analysis was performed using the Infinium CytoSNP-850K BeadChip (SNP-array, Illumina, San Diego, CA, USA) according to the manufacturer’s protocol. Array scanning data were generated on the Illumina iScan System, and the results were analyzed by Bluefuse Multi 4.4 v4 software. Each detected Copy Number Variant was evaluated considering its frequency on healthy human population (DGV, Database of Genomic Variant), gene content, and scientific literature. Next Generation Sequencing (NGS) analysis and variant interpretation: NGS analysis was performed on genomic DNA by using the Twist Custom Panel (clinical exome Twist Bioscience) according to the manufacture’s protocol on Illumina NexSeq550 or NovaSeq6000 platform (Illumina, San Diego, CA, USA). The reads were aligned to human genome build GRCh37/UCSC hg19. The Dragen Enrichment application of BaseSpace (Illumina) and Geneyx Analysis (knowledge-driven NGS Analysis tool powered by the GeneCardsSuite) were used for the variant calling and annotating variants, respectively. Variants of diaphragmatic hernia gene were scored and filtered by the TGex-Geneyx Analysis v6.1 software. Between the variants evaluated by TGex-Geneyx and matching with the “diaphragmatic hernia”, “Marfan”/”Ehlers-Danlos” phenotype. Only the variants meeting all the following parameters were filtered: 1. nonsynonymous exonic or ±5 bp intronic variants; 2. minor allele frequency (MAF) in the Genome Aggregation Database (GnomAD) of less than 0.01 (1%); 3. quality of the call variant: Coverage ≥ 30X and GQ ≥ 50; 4. at least 20% of reads showing the alternative allele (Alt > 20%). Variants were visualized by the Integrative Genome Viewer (IGV). Sequence data were carefully analyzed, and the presence of all suspected variants was checked in the public databases (gnomAD, dbSNP, 1000 Genomes Project, EVS, ExAC). In silico prediction of variants’ pathogenicity was obtained using Sorting Intolerant from Tolerant (SIFT), Polymorphism Phenotyping v2 (PolyPhen-2), and Mutation Taster for the prediction of deleterious non-synonymous SNVs for human diseases. The variants were evaluated by VarSome [24] and classified according to the American College of Medical Genetics and Genomics criteria [25]. The singleton’s variants were confirmed by Sanger sequencing following a standard protocol (BigDye Terminator v3.1 Cycle Sequencing Kit, Applied Biosystems by Life Technologies). Segregation analysis was performed when parents’ samples were available.

### 2.3. Ethical Approval

All Procedures Performed in This Study Were in Accordance with the Ethical Standards of the Institutional and National Research Committee and with the 1964 Helsinki Declaration and Its Later Amendments or Comparable Ethical Standards.

The study was provided by our Scientific Directorate, and it was approved as a retrospective analysis with no patient-identifiable information (RAP 2025-0002, 22 July 2025). The respect of the privacy for personal data of reported patients has been considered according to the specific statement of the Italian Law D. Lgs. n.196 139 of 2003 about personal data protection.

## 3. Results

### 3.1. Clinical Characteristic

Over an eight-year period, we studied 16 patients with joint laxity and positive Beighton scale out of 124 pediatric patients with CDH surgically corrected evaluated during the follow-up program. They include 8/16 (50%) males and 8/16 (50%) females. Mean age at time of genetic testing was 3.9 years (range 2.1–6.3 years). CDH was left-sided in 13/16 (81.3%) patients and right-sided in 3/16 (18.7%). Details about CDH characteristics are shown in Table 1.

Clinical and phenotypical features of the patients are shown in Table 2.

Family history showed no consanguinity. Recurrent CDH was described in one family. Phenotypic characteristics of the proband’s parents are shown in Table 3.

### 3.2. Molecular Results

Variants in the analyzed genes have been identified in 8/16 (50%) patients. Among them, in six patients a single variant was diagnosed, while in two patients the co-occurrence of two variants has been found. All identified variants have been classified as “variants of unknown significance” (vous), according to the American College of Medical Genetics and Genomics criteria [25].

In one case the variant was “de novo”, since it was not found in the parents. In four patients it was inherited from the father, while in five patients, familial segregation was not studied.

Molecular details showing the genes and variants diagnosed in the present series are reported in Table 3. These include: (1) a missense heterozygous “de novo” variant in the *FBN1* gene in one patient, (2) a missense heterozygous paternally inherited variant in *FBN2* gene in one patient, (3) two different missense variants in the *ZNF469* gene in one patient; (4) a frameshift heterozygous variant in the *VEGFA* gene associated with a missense heterozygous variant in the *NOTCH1* gene in the same single patient; (5) a missense heterozygous variant in the *NOTCH1* gene in one patient, (6) a missense heterozygous paternally inherited variant in the *ELN* gene in one patient, (7) a missense heterozygous paternally inherited variant in the *MCTP2* gene in one patient, (8) a missense heterozygous paternally inherited variant in the *SMAD6* gene in one patient.

Clinical characteristics of parents diagnosed with gene variants are shown in Table 3.

## 4. Discussion

The etiology of CDH is unknown in more than 50% of the cases [8], although several heterogeneous causative defects have been identified, including chromosomal anomalies and gene variants, so the role of several pathways has been found.

The diagnosis of heritable connective disorders in patients with CDH has been documented in several instances, prevalently in the setting of Marfan and arterial tortuosity syndromes [14,15]. Different types of diaphragmatic anomalies have been described in Marfan syndrome as diaphragmatic eventration, hiatus/para esophageal hernia, and diaphragmatic hernia. The clinical diagnosis of Marfan syndrome is based on a set of major and minor clinical criteria named the Ghent Nosology [17]. Nevertheless, CDH is not yet listed among clinical criteria in the Ghent nosology of MFS [18].

Connective disorders with joint laxity include, in differential diagnosis, Ehlers-Danlos syndrome, a genetically heterogeneous clinical entity manifesting in skin hyperextensibility and vascular anomalies.

In the present series of subjects with CDH and joint laxity, half of the patients were found to carry variants in analyzed genes, and variants were classified accordingly to the ACMG as vous. For this reason, the variants cannot be considered as surely causative, although the role of predisposition to joint laxity and diaphragmatic hernia cannot be excluded. In addition, the contribution to susceptibility can also be hypothesized since the co-occurrence of variants in two different genes has been documented in one case (a) frameshift variant in *VEGFA* and a missense variant in *NOTCH1*.

In this regard, it is interesting to note that recent studies support a polygenic model for CDH in some instances, at least in European and Latin populations, due to the identification of susceptibility loci for CDH associated with common variants [26].

Parental segregation of the variants in the present series cannot be studied in three families, weakening the evidence of pathogenicity of the variants in these cases.

A “de novo” variant has been detected in a single patient, consisting of the c.4727T>C variant in the *FBN1* gene. The female proband had left CDH associated with joint laxity, slender habitus, arachnodactyly, and patent ductus arteriosus. The diaphragmatic defect was classified as a C size, and the clinical course was complicated by a PH and PDA that was treated by surgery. Marfan-like phenotype was noted during the follow up program when the child was 2 years old.

The *FBN1* gene, causative for Marfan syndrome, is a major component of connective tissue microfibrils glycoprotein and it works as an important calcium binding microfibrillar structural molecule and serves as a regulator of TGF-β signaling involving the structure of elastic and non-elastic tissues. Various anatomic types of CDH have been reported in patients with Marfan syndrome from the literature. The c.4727T>C variant has been described in association with aortic dilation and Marfan-like phenotype, but molecular evidence about pathogenicity is discordant and the variant is at present classified in ClinVar as vous.

A missense heterozygous paternally inherited variant in *FBN2* gene has been diagnosed in a female patient, presenting with relevant joint and skin laxity, pectus excavatum, arachnodactyly, and scoliosis. Her father had a history of scoliosis during childhood without other joint anomalies. The *FBN2* gene is causative for the congenital contractual arachnodactyly also known as Beals syndrome, a connective tissue disorder clinically overlapping with Marfan syndrome but differing for the presence of joint contractures and “crumpled ears” [27].

Two different missense variants in the ZNF469 gene classified as vous have been diagnosed in a patient with mitral valve anomaly, joint laxity, loose skin, and pectus excavatum. The gene is causative for the autosomal recessive Brittle Cornea syndrome 1, also known as Ehlers-Danlos type VIb due to the possible detection of extraocular symptoms as joint and skin laxity, scoliosis, and marfanoid habitus [28].

A vous in *NOTCH1* gene has been detected in two patients, as a single variant in one case and in association with a vous variant in the *VEGFA* gene in another patient. *NOTCH 1* is a member of the Notch signaling pathway and variants in this gene have been reported as causative for left-sided obstructive cardiac lesions (often aortic valve stenosis) and vascular anomalies including aneurism of the ascending aorta and Adams-Oliver syndrome [29]. The *VEGFA* gene is involved in the angiogenesis pathway, and it has been hypothesized that an impaired mechanism of vascularization could play an important role in the pathophysiology of CDH [30]. Both patients had a Marfan-like phenotype and had a diagnosis with CDH size A operated on without complications.

A missense heterozygous paternally inherited variant in the *ELN* gene has been detected in one patient with phenotype of deceivers. The *ELN* gene can be causative for cutis laxa or supravalvular aortic stenosis typical of the Williams syndrome due to chromosome 7q11.23 deletion including *ELN* [31]. Extra skin and extracardiac symptoms can be present in patients with the *ELN* variant, including hernias and lung emphysema. Interestingly, aortic aneurysm and insufficiencies of cardiac valves characteristic for connective tissue disorders have also been reported in patients with *ELN* variants.

A vous variant in *MCTP2* gene has been detected in a patient with Marfan-like phenotype associated with a prenatal CDH type B CDH and agenesis of the pericardium. Surgery was complicated by chylothorax. *MCTP2* has been reported as a dosage-sensitive gene required for cardiac outflow tract development [32]. Rare variants of this gene have been identified, mainly in patients with coarctation of the aorta and bicuspid aortic valve. Further evidence is needed to clarify if a possible link with CDH could exist.

In an additional patient, genetic analysis revealed a vous variant in the *SMAD6* gene, which is reported to be causally related to valvular and vascular defects, including bicuspid aortic valve and thoracic aortic aneurysm [33]. This patient had a postnatal diagnosis of small CDH associated with bicuspid aortic valve.

In regard to the anatomical type of CDH, it is noticeable that left-sided is generally prevalent, and size A according to the Boston classification was diagnosed in the majority of patients with a molecularly detected variant, while size B was prevalently found in patients without identifiable genomic variant.

It should be noted that phenotypical evaluation in toddlers can be difficult since joint laxity and several clinical features characteristics of connective tissue disorders are more evident in follow up controls. In fact, participants ranged from 2 to 6 years of age, so that many phenotypic traits characteristics for connective tissue disorders, including skeletal and cardiovascular anomalies, may not fully manifest at this developmental stage. This could lead to under-detection of connective tissue disorder features. In Table 4, clinical features of patients with detected variants in comparison to major and minor criteria of the Ghent classification for Marfan syndrome [17] and the Villefranche classification for Ehlers-Danlos syndrome [34] have been shown, indicating that aortic dilatation has been diagnosed in a single patient and that ectopia lentis has never been detected in the present group.

Our data underline the importance of a structured clinical follow up program in this group of patients.

Limitations of the study are as follows: (1) A survivorship bias in our series could be due to the fact that our data analysis is referring to the patients born and survived after the surgical intervention for CDH. The study exclusively included CDH patients who survived post-surgical intervention. This potentially excludes more severe or fatal cases and consequently the potential contribution of pathogenic genetic variants in more severe phenotypes may be underestimated; (2) The absence of a control group limits the ability to establish a statistically significant association between genetic variants and clinical manifestations; (3) The small cohort reduces statistical power; (4) The pathogenicity of the identified variants has not been supported by functional assays; (5) The sensitivity and specificity of the Beighton scoring system in toddlers can lead to misclassification bias

## 5. Conclusions

In conclusion: (1) Several “variants of unknown significance” in different genes causative for connective tissue disorders have been detected in half of the present series of patients with CDH and joint laxity; (2) although the majority of the variants are classified accordingly to the ACMG as “variants of unknown significance”, a role of predisposition or susceptibility to CDH cannot be excluded; (3) a precise clinical evaluation for features of connective disorders should be recommended in the diagnostic workflow of patients with CDH.

## Figures and Tables

**Table 1:** Characteristics of diaphragmaic hernia in the present series of patients. Abbreviations: CDH, congenital diaphragmatic hernia; g, grams; F, female; M, male, PH, pulmonary hypertension; PDA, patent ductus arteriosus.

Patient	Sex	Age at Time of Genetic Testing (Years)	CDH Side	CDH Size	Prenatal Diagnosis	Birth Weight (g)	Patch	PH	PDA
1	F	2.7	left	C	yes	2300	yes	yes	yes
2	F	6.2	right	A	no	3650	no	no	yes
3	F	2.1	left	D	yes	3100	yes	yes	no
4	M	6.3	left	A	no	4100	no	no	yes
5	M	4.7	left	A	yes	3780	no	no	yes
6	F	3.5	left	A	yes	2500	no	no	yes
7	F	3.5	left	B	yes	3080	no	no	no
8	M	3.1	left	A	yes	2660	no	no	no
9	M	2.2	left	A	no	2760	no	yes	no
10	F	3.5	left	B	yes	3650	no	no	no
11	M	4.2	left	B	yes	2600	no	no	no
12	M	2.7	left	B	yes	2170	no	yes	no
13	F	3.8	left	B	yes	3300	no	no	no
14	M	6.1	left	D	yes	3200	yes	yes	yes
15	F	4.9	right	B	yes	2780	no	no	no
16	M	2.8	right	B-C	yes	2400	yes	no	no

**Table 2:** Extra diaphragmatic clinical features in the present series of patients.

Patient	Aortic Dilatation	Mitral Anomaly	Joint Laxity	Loose Skin	Marfanoid Habitus	Arachnodactyly	Ectopia Lentis	Pectus Excavatum	Height (Centile)	Additional Features	Beighton Score	Mutant Gene
1	no	no	yes	no	yes	yes	no	no	25th–50th	no	8	*FBN1*
2	no	no	yes	no	yes	yes	no	yes	>97th	no	6	*FBN2*
3	no	yes	yes	yes	no	no	no	yes	50th	intestinal volvulus	5	*ZNF469*
4	no	no	yes	yes	yes	no	no	no	>97th	no	8	*VEGFA NOTCH1*
5	no	yes	yes	no	yes	yes	no	yes	90th–97th	no	7	*NOTCH1*
6	no	no	yes	yes	no	no	no	no	75th	aortic coarctation, aortic arch hypoplasia	6	*ELN*
7	no	no	yes	yes	yes	yes	no	yes	90th–97th	pericardium agenesis	7	*MCTP2*
8	yes	no	yes	no	no	yes	no	no	75th	bicuspid aortic valve	6	*SMAD6*
9	no	no	yes	no	yes	no	no	no	>97th	cognitive deficit	6	-
10	no	no	yes	yes	no	no	no	no	>97th	no	6	-
11	no	no	yes	yes	no	no	no	no	50th–75th	hiatal hernia	5	-
12	no	no	yes	yes	no	no	no	no	90th	no	5	-
13	no	no	yes	yes	no	no	no	no	25th–50th	no	6	-
14	no	yes	no	yes	no	no	no	no	50th	no	5	-
15	no	no	yes	yes	no	no	no	no	50th–75th	no	6	-
16	no	no	yes	no	no	no	no	no	50th–75th	intestinal volvulus	5	-

**Table 3:** Molecular characteristics in the present series of patients with variant in analyzed genes.

Patient	Gene	Variant HGVS	Allelic Freq. (GnomAD)	ACMG	Reference (PMID)	Segregation	Parental Phenotype	Familial CDH	SNParray
1	*FBN1*	NM_000138.5:c.4727T>Cp.(Met1576Thr)	0.000107	VUS	24833718, 31211626	de novo	normal	no	arr[GRCh37] 15q11.2(22753733_23226254)x3 mat
2	*FBN2*	NM_001999.4):c.976C>T p.(Pro326Ser)	0.0067	LB	17935258	paternal	Scoliosis, articular problems in childhood	no	WT
3	*ZNF469* *ZNF469*	NM_*001127464*.2: c.6095C>A (p.Ser2032Tyr) NM_001127464.2: c.4372G>A (p.Asp1458Asn)	- -	VUS VUS	- -	na	normal	no	arr[GRCh37] 14q21.1(42204916_42438099)x1
4	*VEGFA* *NOTCH1*	NM_003376.6):c.19_22del p.(Asp7ProfsTer45) NM_017617.5:c.1295C>T p.(Thr432Met)	- 0.000202	LP LB	- -	na	normal	no	WT
5	*NOTCH1*	NM_017617.5:c.520A>G p.(Thr174Ala)	-	VUS	-	na	normal	no	arr[GRCh37] 22q12.3(32782128_32978251)x3
6	*ELN*	NM_000501.4:c.1909G>T p.(Ala637Ser)	0.0000266	VUS	-	paternal	joint laxity	no	na
7	*MCTP2*	NM_018349.4:c.1591G>C p.(Asp531His)	0.00000399	VUS	-	paternal	tall stature	no	WT
8	*SMAD6*	NM_005585.5:c.391G>A p.(Glu131Lys)	-	VUS	-	paternal	normal	no	WT

Abbreviations: ACMG (American College of Medical Genetics and Genomic); CDH (Congenital Diaphragmatic Hernia); LP, likely pathogenetic; mat, maternal; VUS (Variant of Unknown Significance); na (not available); LB (Likely Benign).

**Table 4:** Extradiaphragmatic clinical features of the patients with detected gene variant in comparison to major and minor clinical signs included in the Ghent criteria for Marfan syndrome and in the Villafranche criteria for classic Ehlers-Danlos syndrome.

Ghent Criteria for Marfan Syndrome	Patient 1	Patient 2	Patient 3	Patient 4	Patient 5	Patient 6	Patient 7	Patient 8
Gene	*FBN1*	*FBN2*	*ZNF469*	*VEGFA* *NOTCH1*	*NOTCH1*	*ELN*	*MCTP2*	*SMAD6*
Major criteria								
Aortic root dilatation with a Z-score of 2 or more	no	no	no	no	no	no	no	yes
Ectopia lentis	no	no	no	no	no	no	no	no
Minor criteria								
Wrist and/or thumb sign	yes	yes	yes	no	yes	no	yes	yes
Pectus carinatum or excavatum	no	yes	yes	no	yes	no	yes	no
Hindfoot deformity	no	no	yes	yes	no	no	yes	no
Pneumothorax	no	no	no	no	no	no	no	no
Dural ectasia	NK	NK	NK	NK	NK	NK	NK	NK
Protrusio acetabuli	NK	NK	NK	NK	NK	NK	NK	NK
Reduced US/LS and increased arm/height	yes	yes	no	yes	yes	no	yes	yes
Scoliosis or thoracolumbar kyphosis	no	no	no	no	no	no	no	no
Reduced elbow extension	no	yes	no	no	no	no	no	no
Facial features	yes	yes	no	yes	yes	yes	no	no
Skin striae	no	no	no	no	no	no	no	no
Myopia > 3 diopters	no	no	no	no	no	no	no	no
Mitral valve prolapse	no	no	yes	no	yes	no	no	no
Villefranche criteria for classical Ehlers-Danlos syndrome								
Major criteria								
Skin hyperextensibility	no	no	yes	yes	no	yes	yes	no
Widened atrophic scars (manifestation of tissue fragility)	no	no	no	no	no	no	no	no
Joint hypermobility	yes	yes	yes	yes	yes	yes	yes	yes
Minor criteria								
Smooth velvety skin	no	no	yes	no	no	yes	yes	no
Molluscoid pseudotumours	no	no	no	no	no	no	no	no
Subcutaneous spheroids/spherules	no	no	no	no	no	no	no	no
Complications of joint hypermobility	yes	yes	yes	no	yes	no	no	yes
Muscle hypotonia, delayed gross motor development	yes	yes	no	no	yes	no	no	no
Easy bruising	no	no	no	no	no	no	no	no
Manifestations of tissue extensibility and fragility	no	no	yes	no	no	no	no	no
Surgical complications (postoperative hernias)	no	no	no	no	no	no	no	no
Positive family history	no	yes	no	no	no	yes	yes	no

## Data Availability

The datasets used and/or analyzed during the current study are available from the corresponding author upon reasonable request for research only.
